# Crystal structure of 1-tosyl-1,2,3,4-tetra­hydro­quinoline

**DOI:** 10.1107/S1600536814022181

**Published:** 2014-10-24

**Authors:** S. Jeyaseelan, K.V. Asha, G. Venkateshappa, P. Raghavendrakumar, B.S. Palakshamurthy

**Affiliations:** a Department of Physics, St Philomena’s College (Autonomous), Mysore, karnataka 570 015, India; b Department of Studies and Research in Physics, UCS, Tumkur University, Karnataka 572 103, India; c Department of Chemistry, UCST, Tumkur University, Karnataka 572 103, India

**Keywords:** crystal structure, quinolines, C—H⋯O inter­actions, biotransformations, pharmacological activity

## Abstract

In the title compound, C_16_H_17_NO_2_S, the heterocyclic ring adopts a half-chair conformation and the bond-angle sum at the N atom is 350.2°. The dihedral angle between the planes of the aromatic rings is 47.74 (10)°. In the crystal, mol­ecules are linked by C—H⋯O hydrogen bonds to generate [010] chains.

## Related literature   

For reactions related to biotransformations, see: Leresche *et al.* (2006 ▶); Astudillo *et al.* (2009 ▶). For pharmacological activities, see: Bendale *et al.* (2007 ▶); Chen *et al.* (2007 ▶); Singer *et al.* (2005 ▶).
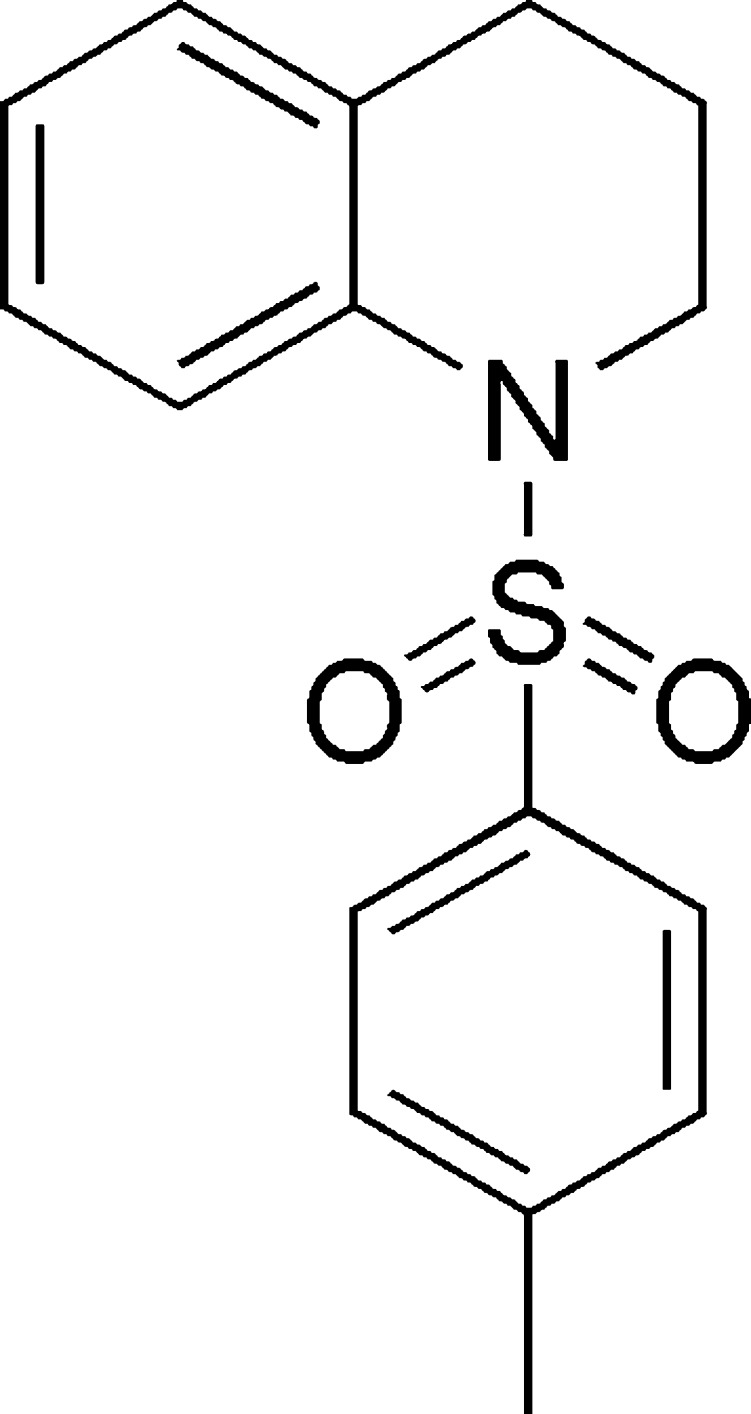


## Experimental   

### Crystal data  

C_16_H_17_NO_2_S
*M* *_r_* = 287.37
Monoclinic, 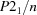

*a* = 8.2176 (7) Å
*b* = 8.0468 (6) Å
*c* = 22.2439 (18) Å
β = 98.107 (4)°
*V* = 1456.2 (2) Å^3^
*Z* = 4
Mo *K*α radiation
μ = 0.22 mm^−1^
*T* = 94 K
0.24 × 0.22 × 0.18 mm

### Data collection  

Bruker APEXII CCD diffractometer
Absorption correction: multi-scan (*SADABS*; Bruker, 2013 ▶) *T*_min_ = 0.949, *T*_max_ = 0.961
20017 measured reflections
2568 independent reflections
2327 reflections with *I* > 2σ(*I*)
*R*_int_ = 0.046

### Refinement  

*R*[*F* ^2^ > 2σ(*F* ^2^)] = 0.039
*wR*(*F* ^2^) = 0.101
*S* = 1.09
2568 reflections
182 parameters
H-atom parameters constrained
Δρ_max_ = 0.23 e Å^−3^
Δρ_min_ = −0.37 e Å^−3^

### 

Data collection: *APEX2* (Bruker, 2013 ▶); cell refinement: *SAINT* (Bruker, 2013 ▶); data reduction: *SAINT*; program(s) used to solve structure: *SHELXS97* (Sheldrick, 2008 ▶); program(s) used to refine structure: *SHELXL97* (Sheldrick, 2008 ▶); molecular graphics: *ORTEP-3 for Windows* (Farrugia, 2012 ▶) and *Mercury* (Macrae *et al.*, 2008 ▶); software used to prepare material for publication: *SHELXL97*.

## Supplementary Material

Crystal structure: contains datablock(s) I. DOI: 10.1107/S1600536814022181/hb7292sup1.cif

Structure factors: contains datablock(s) I. DOI: 10.1107/S1600536814022181/hb7292Isup2.hkl

Supporting information file. DOI: 10.1107/S1600536814022181/hb7292Isup3.cml

**Fig. 1.**
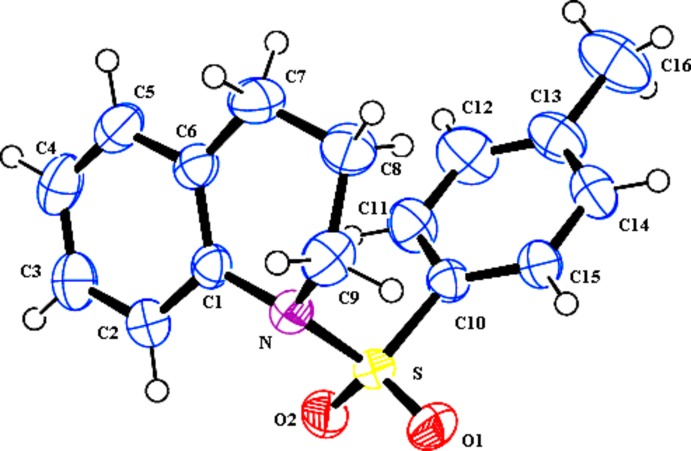
The molecular structure of the title compound, showing displacement ellipsoids drawn at the 50% probability level.

. DOI: 10.1107/S1600536814022181/hb7292fig1.tif
The mol­ecular structure of the title compound, showing displacement ellipsoids drawn at the 50% probability level.

**Fig. 2.**
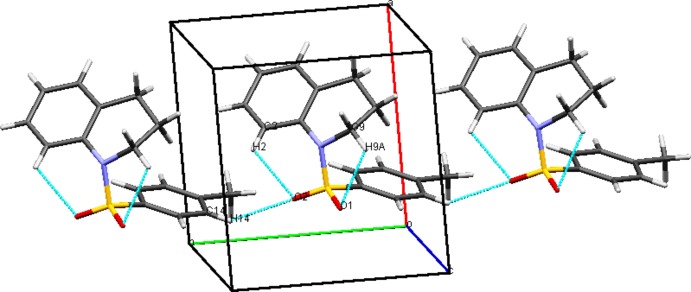
The molecular packing of the title compound, dashed lines indicate intramolecular C—H···O and intermolecular C—H···O hydrogen bonds forming C(6) chains viewed along [010].

C . DOI: 10.1107/S1600536814022181/hb7292fig2.tif
The mol­ecular packing of the title compound, dashed lines indicate intra­molecular C—H⋯O and inter­molecular C—H⋯O hydrogen bonds forming *C*(6) chains viewed along [010].

CCDC reference: 1028050

Additional supporting information:  crystallographic information; 3D view; checkCIF report

## Figures and Tables

**Table 1:** Hydrogen-bond geometry (, )

*D*H*A*	*D*H	H*A*	*D* *A*	*D*H*A*
C14H14O2^i^	0.95	2.53	3.340(2)	143

Symmetry code: (i) 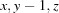.

## supplementary crystallographic information

### S1. Chemical context

Chemical reactions by biotransformations have a number of advantages because they play an important role in the production of chiral products from racemic mixtures (Leresche *et al.*, 2006) in perticlar the tetra­hydro­quinoline derivatives can be transformed to other by *Mortierella isabelina* (Astudillo *et al.*, 2009). The tetra­hydro­quinoline compounds are core structures in pharmacological activities such as anti­malarial activities (Bendale *et al.*, 2007), anti­psychotic (Singer *et al.*, 2005), estrogenic receptors (Chen *et al.*, 2007). In the course of our study, we noticed that 1,2,3,4-tetra­hydro­quinoline derivatives exhibit a few pharmacological activities (our unpublished data). As a part of our study we have undertaken crystal structure determination of the title compound and the results are presented here.

### S2. Structural commentary

The molecular structure of the title compound is shown in Fig. 1. In the title molecule, the planes of the C1–C6 and C10–C15 benzene rings form a dihedral angle of 47.74 (9)°. The C1/C6–C9/N1 ring is in a half-chair conformation, with the methyl­ene C9 atom as the flap. The molecular structure is stabilized by intra­molecular C9—H9A···O1 and C2—H2···O2 hydrogen bonds (Fig. 2).

### S3. Supra­molecular features

In the crystal structure, inter­molecular C14—H14···O2 hydrogen bonds link molecules into *C*(6) chains along [010] (Fig. 2 and Table 1)

### S5. Synthesis and crystallization

To a stirred solution of 1,2,3,4-tetra­hydro­quinoline (10 mmol) in 30 mL dry di­chloro­ethane, tri­ethyl­amine (15 mmol) was added at 0 – 5°C. To this reaction mixture 4-methyl­benzene-1-sulfonyl­chloride (12 mmol) was added drop wise. After 2h of stirring at room temperature, the reaction mixture was washed with 5% Na_2_CO_3_ and brine. Organic phase was dried over Na_2_SO_4_ and then it was concentrated on vacuum to yield titled compound as colourless solid. The crude product was recrystallized in the mixture of ethyl acetate and hexane­(1:1) to get colourless prisms.

### S6. Refinement

Crystal data, data collection and structure refinement details are summarized in Table 1. The H atoms were positioned with idealized geometry using a riding model with C—H = 0.95-0.99 Å. All H-atoms were refined with isotropic displacement parameters (set to 1.2-1.5 times of the U eq of the parent atom).

### Crystal data

**Table:**

C_16_H_17_NO_2_S	Prism
*M**_r_* = 287.37	*D*_x_ = 1.311 Mg m^−^^3^
Monoclinic, *P*2_1_/*n*	Melting point: 402 K
Hall symbol: -P 2yn	Mo *K*α radiation, λ = 0.71073 Å
*a* = 8.2176 (7) Å	Cell parameters from 2327 reflections
*b* = 8.0468 (6) Å	θ = 1.9–25.0°
*c* = 22.2439 (18) Å	µ = 0.22 mm^−^^1^
β = 98.107 (4)°	*T* = 94 K
*V* = 1456.2 (2) Å^3^	Prism, colourless
*Z* = 4	0.24 × 0.22 × 0.18 mm
*F*(000) = 608	

### Data collection

**Table:**

Bruker APEXII CCD diffractometer	2568 independent reflections
Radiation source: fine-focus sealed tube	2327 reflections with *I* > 2σ(*I*)
Graphite monochromator	*R*_int_ = 0.046
Detector resolution: 1.9 pixels mm^-1^	θ_max_ = 25.0°, θ_min_ = 1.9°
phi and ω scans	*h* = −9→9
Absorption correction: multi-scan (*SADABS*; Bruker, 2013)	*k* = −9→9
*T*_min_ = 0.949, *T*_max_ = 0.961	*l* = −26→26
20017 measured reflections	

### Refinement

**Table:**

Refinement on *F*^2^	Primary atom site location: structure-invariant direct methods
Least-squares matrix: full	Secondary atom site location: difference Fourier map
*R*[*F*^2^ > 2σ(*F*^2^)] = 0.039	Hydrogen site location: inferred from neighbouring sites
*wR*(*F*^2^) = 0.101	H-atom parameters constrained
*S* = 1.09	*w* = 1/[σ^2^(*F*_o_^2^) + (0.0362*P*)^2^ + 0.8395*P*] where *P* = (*F*_o_^2^ + 2*F*_c_^2^)/3
2568 reflections	(Δ/σ)_max_ = 0.001
182 parameters	Δρ_max_ = 0.23 e Å^−^^3^
0 restraints	Δρ_min_ = −0.37 e Å^−^^3^
0 constraints	

### Special details

**Table:**

Geometry. All e.s.d.'s (except the e.s.d. in the dihedral angle between two l.s. planes) are estimated using the full covariance matrix. The cell e.s.d.'s are taken into account individually in the estimation of e.s.d.'s in distances, angles and torsion angles; correlations between e.s.d.'s in cell parameters are only used when they are defined by crystal symmetry. An approximate (isotropic) treatment of cell e.s.d.'s is used for estimating e.s.d.'s involving l.s. planes.
Refinement. Refinement of *F*^2^ against ALL reflections. The weighted *R*-factor *wR* and goodness of fit *S* are based on *F*^2^, conventional *R*-factors *R* are based on *F*, with *F* set to zero for negative *F*^2^. The threshold expression of *F*^2^ > σ(*F*^2^) is used only for calculating *R*-factors(gt) *etc*. and is not relevant to the choice of reflections for refinement. *R*-factors based on *F*^2^ are statistically about twice as large as those based on *F*, and *R*- factors based on ALL data will be even larger.

### Fractional atomic coordinates and isotropic or equivalent isotropic displacement parameters (Å^2^)

**Table:**

	*x*	*y*	*z*	*U*_iso_*/*U*_eq_	
C1	0.5482 (2)	0.4736 (2)	0.17137 (8)	0.0359 (4)	
C2	0.5261 (3)	0.6396 (2)	0.15480 (9)	0.0461 (5)	
H2	0.4386	0.7015	0.1675	0.055*	
C3	0.6312 (3)	0.7140 (3)	0.12003 (10)	0.0565 (6)	
H3	0.6146	0.8265	0.1078	0.068*	
C4	0.7605 (3)	0.6253 (3)	0.10294 (10)	0.0593 (6)	
H4	0.8319	0.6758	0.0782	0.071*	
C5	0.7859 (2)	0.4634 (3)	0.12175 (9)	0.0509 (5)	
H5	0.8772	0.4043	0.1106	0.061*	
C6	0.6818 (2)	0.3838 (2)	0.15666 (8)	0.0400 (4)	
C7	0.7232 (3)	0.2110 (3)	0.18038 (11)	0.0553 (6)	
H7A	0.7451	0.1398	0.1461	0.066*	
H7B	0.8255	0.2161	0.2098	0.066*	
C8	0.5908 (3)	0.1313 (3)	0.21082 (11)	0.0553 (6)	
H8A	0.6394	0.0426	0.2386	0.066*	
H8B	0.5079	0.0799	0.1798	0.066*	
C9	0.5085 (3)	0.2589 (3)	0.24637 (9)	0.0505 (5)	
H9A	0.4200	0.2043	0.2651	0.061*	
H9B	0.5900	0.3033	0.2795	0.061*	
C10	0.2228 (2)	0.2132 (2)	0.12881 (8)	0.0368 (4)	
C11	0.2794 (3)	0.2300 (3)	0.07339 (9)	0.0536 (5)	
H11	0.3210	0.3333	0.0616	0.064*	
C12	0.2741 (4)	0.0936 (3)	0.03583 (10)	0.0661 (7)	
H12	0.3131	0.1040	−0.0022	0.079*	
C13	0.2137 (3)	−0.0582 (3)	0.05179 (10)	0.0577 (6)	
C14	0.1579 (3)	−0.0714 (3)	0.10703 (10)	0.0524 (5)	
H14	0.1156	−0.1745	0.1187	0.063*	
C15	0.1626 (2)	0.0630 (2)	0.14573 (9)	0.0427 (4)	
H15	0.1244	0.0520	0.1839	0.051*	
C16	0.2090 (4)	−0.2057 (4)	0.00972 (13)	0.0920 (10)	
H16A	0.2151	−0.3085	0.0336	0.138*	
H16B	0.3025	−0.2004	−0.0131	0.138*	
H16C	0.1062	−0.2042	−0.0186	0.138*	
O1	0.15775 (18)	0.33842 (19)	0.22946 (7)	0.0548 (4)	
O2	0.19664 (17)	0.52807 (17)	0.14578 (7)	0.0532 (4)	
N	0.43800 (18)	0.39748 (19)	0.20781 (7)	0.0381 (4)	
S	0.24138 (5)	0.38103 (6)	0.17987 (2)	0.03916 (16)	

### Atomic displacement parameters (Å^2^)

**Table:**

	*U*^11^	*U*^22^	*U*^33^	*U*^12^	*U*^13^	*U*^23^
C1	0.0337 (9)	0.0389 (10)	0.0349 (9)	−0.0051 (8)	0.0038 (7)	−0.0037 (8)
C2	0.0459 (11)	0.0401 (11)	0.0527 (11)	−0.0041 (9)	0.0082 (9)	−0.0020 (9)
C3	0.0605 (14)	0.0486 (12)	0.0607 (13)	−0.0124 (11)	0.0102 (11)	0.0080 (10)
C4	0.0523 (13)	0.0752 (16)	0.0527 (12)	−0.0209 (12)	0.0152 (10)	0.0045 (12)
C5	0.0367 (11)	0.0699 (15)	0.0470 (11)	−0.0057 (10)	0.0092 (9)	−0.0098 (11)
C6	0.0341 (10)	0.0479 (11)	0.0373 (9)	−0.0024 (8)	0.0025 (8)	−0.0061 (8)
C7	0.0451 (12)	0.0545 (13)	0.0660 (14)	0.0119 (10)	0.0070 (10)	−0.0018 (11)
C8	0.0496 (12)	0.0458 (12)	0.0689 (14)	0.0112 (10)	0.0029 (10)	0.0139 (10)
C9	0.0495 (12)	0.0565 (13)	0.0457 (11)	0.0021 (10)	0.0074 (9)	0.0137 (10)
C10	0.0365 (10)	0.0350 (10)	0.0381 (9)	0.0010 (8)	0.0028 (7)	0.0014 (8)
C11	0.0704 (15)	0.0471 (12)	0.0444 (11)	−0.0039 (11)	0.0118 (10)	0.0067 (9)
C12	0.0952 (19)	0.0675 (16)	0.0370 (11)	0.0029 (14)	0.0138 (12)	−0.0015 (11)
C13	0.0697 (15)	0.0497 (13)	0.0498 (12)	0.0089 (11)	−0.0056 (11)	−0.0092 (10)
C14	0.0595 (13)	0.0374 (11)	0.0578 (13)	−0.0024 (10)	−0.0002 (10)	0.0005 (9)
C15	0.0454 (11)	0.0390 (10)	0.0439 (10)	−0.0020 (8)	0.0066 (9)	0.0028 (8)
C16	0.127 (3)	0.0716 (19)	0.0739 (18)	0.0083 (18)	0.0008 (18)	−0.0298 (15)
O1	0.0478 (8)	0.0610 (9)	0.0612 (9)	−0.0083 (7)	0.0268 (7)	−0.0128 (7)
O2	0.0399 (8)	0.0352 (7)	0.0831 (11)	0.0066 (6)	0.0040 (7)	0.0050 (7)
N	0.0361 (8)	0.0379 (8)	0.0410 (8)	−0.0006 (7)	0.0084 (7)	0.0007 (7)
S	0.0332 (3)	0.0345 (3)	0.0516 (3)	0.00026 (18)	0.0119 (2)	−0.0035 (2)

### Geometric parameters (Å, º)

**Table:**

C1—C2	1.390 (3)	C10—C15	1.378 (3)
C1—C6	1.392 (3)	C10—C11	1.384 (3)
C1—N	1.435 (2)	C10—S	1.7577 (19)
C2—C3	1.375 (3)	C11—C12	1.377 (3)
C2—H2	0.9500	C11—H11	0.9500
C3—C4	1.377 (3)	C12—C13	1.384 (3)
C3—H3	0.9500	C12—H12	0.9500
C4—C5	1.375 (3)	C13—C14	1.374 (3)
C4—H4	0.9500	C13—C16	1.508 (3)
C5—C6	1.390 (3)	C14—C15	1.379 (3)
C5—H5	0.9500	C14—H14	0.9500
C6—C7	1.509 (3)	C15—H15	0.9500
C7—C8	1.504 (3)	C16—H16A	0.9800
C7—H7A	0.9900	C16—H16B	0.9800
C7—H7B	0.9900	C16—H16C	0.9800
C8—C9	1.512 (3)	O1—S	1.4213 (14)
C8—H8A	0.9900	O2—S	1.4254 (14)
C8—H8B	0.9900	N—S	1.6526 (16)
C9—N	1.475 (2)	S—O1	1.4213 (14)
C9—H9A	0.9900	S—O2	1.4254 (14)
C9—H9B	0.9900		
			
C2—C1—C6	120.95 (17)	H9A—C9—H9B	107.9
C2—C1—N	119.39 (17)	C15—C10—C11	120.52 (18)
C6—C1—N	119.52 (17)	C15—C10—S	119.94 (14)
C3—C2—C1	119.9 (2)	C11—C10—S	119.40 (15)
C3—C2—H2	120.1	C12—C11—C10	118.5 (2)
C1—C2—H2	120.1	C12—C11—H11	120.8
C2—C3—C4	120.0 (2)	C10—C11—H11	120.8
C2—C3—H3	120.0	C11—C12—C13	122.0 (2)
C4—C3—H3	120.0	C11—C12—H12	119.0
C5—C4—C3	119.8 (2)	C13—C12—H12	119.0
C5—C4—H4	120.1	C14—C13—C12	118.3 (2)
C3—C4—H4	120.1	C14—C13—C16	120.8 (2)
C4—C5—C6	121.8 (2)	C12—C13—C16	120.9 (2)
C4—C5—H5	119.1	C13—C14—C15	121.0 (2)
C6—C5—H5	119.1	C13—C14—H14	119.5
C5—C6—C1	117.40 (19)	C15—C14—H14	119.5
C5—C6—C7	119.59 (18)	C10—C15—C14	119.75 (19)
C1—C6—C7	122.88 (17)	C10—C15—H15	120.1
C8—C7—C6	114.12 (17)	C14—C15—H15	120.1
C8—C7—H7A	108.7	C13—C16—H16A	109.5
C6—C7—H7A	108.7	C13—C16—H16B	109.5
C8—C7—H7B	108.7	H16A—C16—H16B	109.5
C6—C7—H7B	108.7	C13—C16—H16C	109.5
H7A—C7—H7B	107.6	H16A—C16—H16C	109.5
C7—C8—C9	110.59 (19)	H16B—C16—H16C	109.5
C7—C8—H8A	109.5	C1—N—C9	115.09 (15)
C9—C8—H8A	109.5	C1—N—S	118.90 (12)
C7—C8—H8B	109.5	C9—N—S	116.17 (13)
C9—C8—H8B	109.5	O1—S—O2	119.76 (9)
H8A—C8—H8B	108.1	O1—S—N	106.36 (9)
N—C9—C8	112.16 (16)	O2—S—N	107.38 (8)
N—C9—H9A	109.2	O1—S—C10	107.98 (9)
C8—C9—H9A	109.2	O2—S—C10	107.57 (9)
N—C9—H9B	109.2	N—S—C10	107.19 (8)
C8—C9—H9B	109.2		

### Hydrogen-bond geometry (Å, º)

**Table:**

*D*—H···*A*	*D*—H	H···*A*	*D*···*A*	*D*—H···*A*
C14—H14···O2^i^	0.95	2.53	3.340 (2)	143

Symmetry code: (i) *x*, *y*−1, *z*.
