# Supplementary figures and images for: Crystal structure of 1-tosyl-1,2,3,4-tetra­hydro­quinoline
Source: Acta Crystallogr Sect E Struct Rep Online. 2014 Oct 24;70(Pt 11):o1176. doi: 10.1107/S1600536814022181 (PMC4257240; doi:10.1107/S1600536814022181)

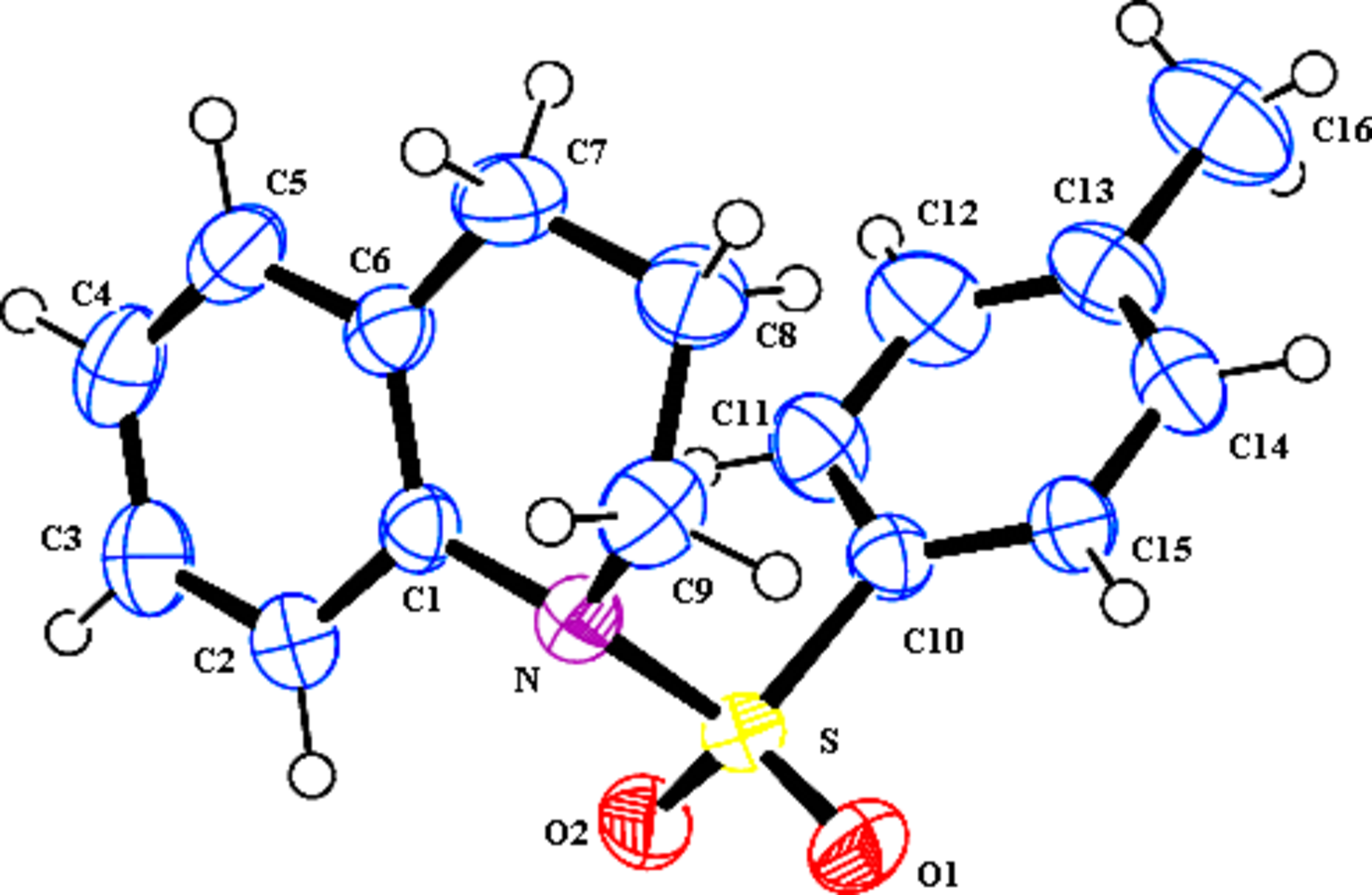

Supplement: Supplementary file 4 [file e-70-o1176-fig1.tif]

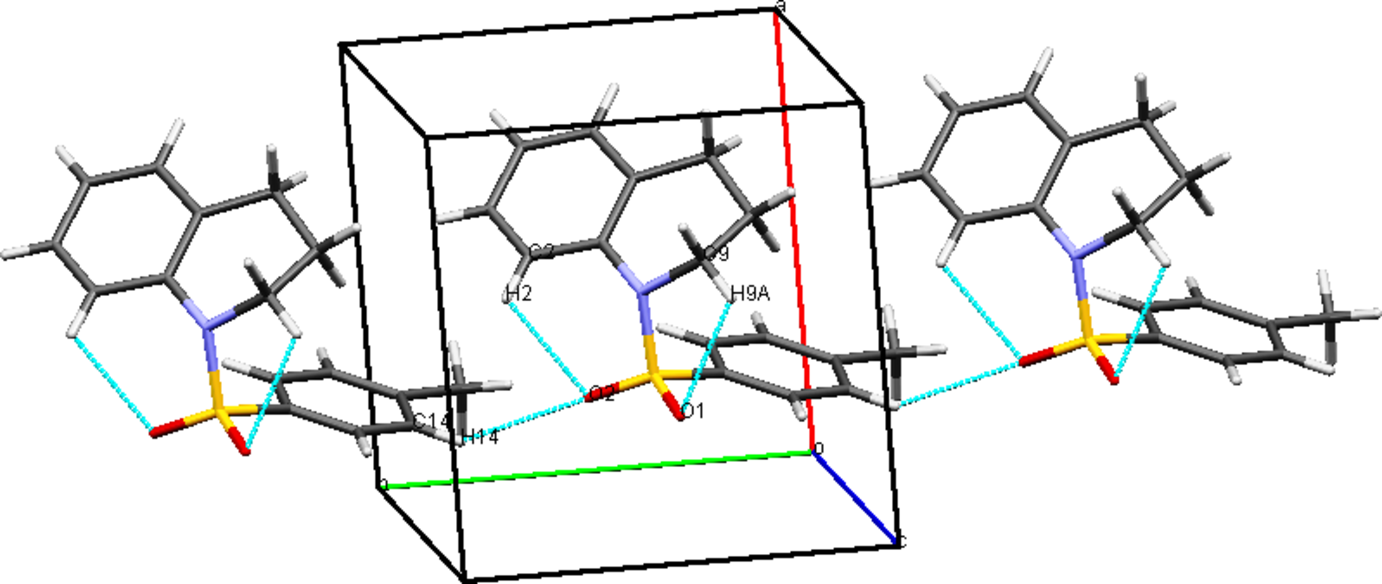

Supplement: Supplementary file 5 [file e-70-o1176-fig2.tif]
